# Saporin-S6: A Useful Tool in Cancer Therapy

**DOI:** 10.3390/toxins5101698

**Published:** 2013-10-07

**Authors:** Letizia Polito, Massimo Bortolotti, Daniele Mercatelli, Maria Giulia Battelli, Andrea Bolognesi

**Affiliations:** Department of Experimental, Diagnostic and Specialty Medicine-DIMES; Alma Mater Studiorum, University of Bologna, Via San Giacomo 14, 40126 Bologna, Italy; E-Mails: massimo.bortolotti2@unibo.it (M.B.); daniele.mercatelli2@unibo.it (D.M.); mariagiulia.battelli@unibo.it (M.G.B.); andrea.bolognesi@unibo.it (A.B.)

**Keywords:** saporin-S6, saporin, *Saponaria officinalis*, immunotoxin, immunotherapy, ribosome-inactivating proteins, monoclonal antibodies, rRNA *N*-glycosylase activity, anti-cancer therapy, hematological tumors.

## Abstract

Thirty years ago, the type 1 ribosome-inactivating protein (RIP) saporin-S6 (also known as saporin) was isolated from *Saponaria officinalis* L. seeds. Since then, the properties and mechanisms of action of saporin-S6 have been well characterized, and it has been widely employed in the construction of conjugates and immunotoxins for different purposes. These immunotoxins have shown many interesting results when used in cancer therapy, particularly in hematological tumors. The high enzymatic activity, stability and resistance to conjugation procedures and blood proteases make saporin-S6 a very useful tool in cancer therapy. High efficacy has been reported in clinical trials with saporin-S6-containing immunotoxins, at dosages that induced only mild and transient side effects, which were mainly fever, myalgias, hepatotoxicity, thrombocytopenia and vascular leak syndrome. Moreover, saporin-S6 triggers multiple cell death pathways, rendering impossible the selection of RIP-resistant mutants. In this review, some aspects of saporin-S6, such as the chemico-physical characteristics, the structural properties, its endocytosis, its intracellular routing and the pathogenetic mechanisms of the cell damage, are reported. In addition, the recent progress and developments of saporin-S6-containing immunotoxins in cancer immunotherapy are summarized, including *in vitro* and *in vivo* pre-clinical studies and clinical trials.

## 1. Overview

Saporin-S6 (also known as saporin) is a plant toxin belonging to the ribosome-inactivating protein (RIP) family, a class of toxic enzymes that is widely distributed among plant genera whose activity is classically identified as rRNA *N*-glycosylase (EC 3.2.2.22). RIPs specifically remove the A4324 adenine residue, which forms part of a tetranucleotide G(A4324)GA sequence on the ricin/sarcin region of the 28S rRNA in the 60S subunit of the rat ribosome, a sequence that is universally conserved among eukaryotic rRNA. Adenine removal interferes with the interaction between the ribosome and the elongation factor 2, damaging ribosomes in an irreversible manner and causing the inhibition of protein synthesis [1]. RIPs also show *in vitro N*-glycosylase activity on different substrates, such as mRNA, tRNA, DNA and poly(A) [2,3], and some RIPs have also shown activity against poly(ADP-ribosyl)ated proteins [4]. For this reason, the enzymatic activity of RIPs has been defined as polynucleotide:adenosine glycosylase (PNAG) [3].

RIPs are classified into type 1, which consist of a single-chain protein with enzymatic activity, type 2, with an enzymatic A-chain linked by a disulfide bond to a lectin B-chain that is able to bind to sugar-containing cell surface receptors, and type 3, composed of an *N*-terminal catalytic domain and an extended *C*-terminal domain whose function is unknown [5]. The presence of the B-chain in type 2 RIPs allows the fast internalization of the toxin into the cell. Inside the cell, the A-B moieties are separated so that the active A-chain can enter the cytosol, where it exerts its ribotoxic action. For this reason, most type 2 RIPs are very toxic. However, a number of non-toxic type 2 RIPs were found in some plant species belonging to the *Sambucus* genus [6]. Despite the differences reported for animal and cell toxicity [7], RIPs often show a similar activity on ribosomes in a cell-free system [8].

RIPs have been widely studied because of their potential therapeutic application in a variety of human diseases as toxic moiety of a conjugate. The conjugation of a cytotoxic RIP to a target-specific carrier, such as a monoclonal antibody (mAb), allows the selective killing of target cells. Conjugates containing antibodies or their fragments are referred to as immunotoxins (ITs). ITs have been obtained both by the chemical linkage of the toxic moiety to mAbs and by genetic engineering to obtain recombinant conjugates [9]. RIP-containing ITs have been included in clinical trials against various diseases, often achieving promising results, especially in the treatment of hematological neoplasms [10]. A series of ITs containing different Abs and type 1 RIPs has been previously described with very interesting results also in clinical trials [11,12]. The use of type 1 RIPs to construct ITs have several advantages: they are stable, safe to handle, numerous and often immunologically not correlated. Moreover, the wide variety of type 1 RIPs allows to select proteins with different characteristics, such as low systemic toxicity, high stability, *etc*.

This review summarizes the chemico-physical and structural properties of saporin-S6 and the characteristics of its endocytosis and intracellular routing. The pathogenetic mechanisms of the cell damage mediated by saporin-S6 are also described. Moreover, the recent progress and developments of saporin-S6-containing ITs in cancer immunotherapy are reported, considering both pre-clinical studies and clinical trials. 

## 2. Saporin-S6

### 2.1. Chemico-Physical and Structural Properties

**Figure 1:**
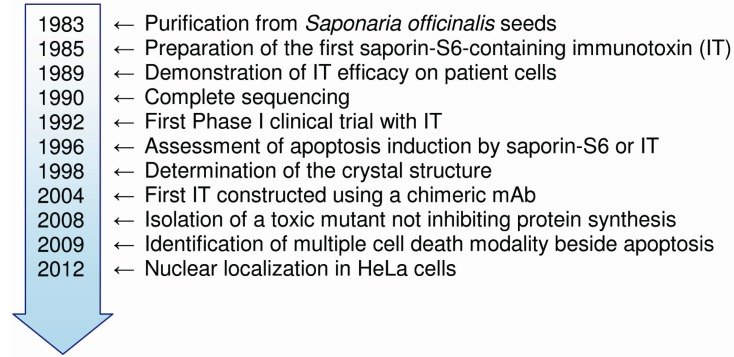
Chronological advancements in the research on saporin-S6. Each reference is listed in the appropriate section of the main text.

The first description of type 1 RIPs in soapwort seeds (*Saponaria officinalis*, Caryophyllaceae family) dates back three decades [13]. Since then, saporin-S6 has been the protagonist of more than a thousand studies reported in literature. Some important steps in the saporin-S6 timeline are reported in Figure 1.

Saporin-S6 belongs to a multigene family of proteins that includes more than nine different isoforms isolated from various plant tissues, such as leaf, root, and seed [14]. All isoforms differ from each other in both their chemico-physical and biological properties, but they all have a molecular weight of approximately 30 kDa. Saporin-S6 is the most representative of the seed isoforms, accounting for approximately 7% of the total seed protein content [13]. Saporin-S6 sequencing revealed heterogeneity at two positions, with either aspartic or glutamic acid in position 48, and either lysine or arginine present in position 91, which indicates that the saporin-S6 chromatographic peak contains a set of closely related saporin isoforms [15,16]. Notably, saporin-S6 is one of the most studied among the type 1 RIPs because of its strong activity both in cell-free systems and in cell lines. 

The mature form of saporin-S6 is 253 amino acids long. The sequence was determined in 1990 and almost 10% of the amino acids in saporin-S6 are lysine residues, a condition that confers to the protein an extremely high p*I* (approximately 10) [15]. No neutral sugars are present in the saporin-S6 molecule, despite the presence of glycosylation sites in the pro-saporin *C*-terminal extension sequence that is cleaved to form the mature protein. 

**Figure 2:**
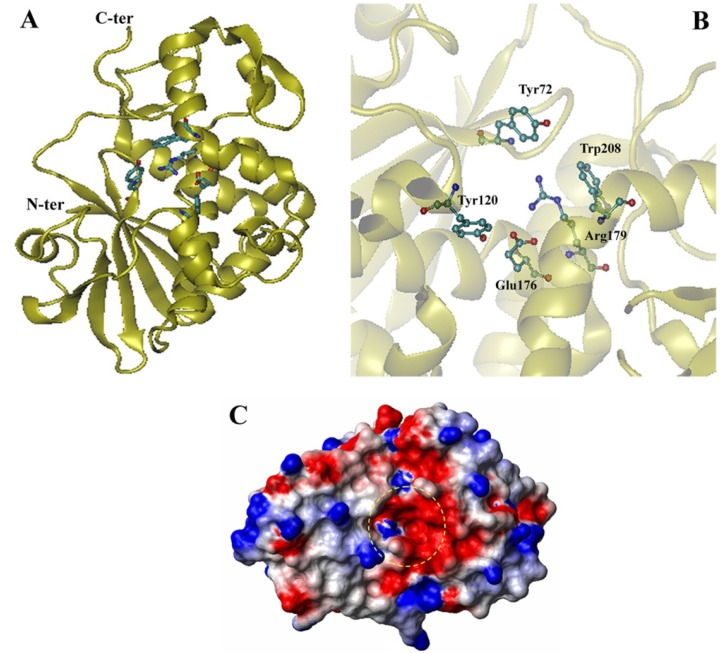
Structural characteristics of saporin-S6. Ribbon model of the crystal structure (PDB 1QI7) (**A**) and catalytic site (**B**) of saporin-S6. The key residues of the enzymatic site are presented using a ball-and-stick model. Figures were produced by VMD 1.9.1 software. Electrostatic potential (**C**) of the saporin-S6 surface at pH 7. The positive (blue) and the negative (red) regions are shown. The active pocket is highlighted by a yellow circle. The image was produced using the MOLMOL program.

The 2.0 Å resolution crystal structure of saporin-S6 showed that this protein contains two main domains: a predominantly β-stranded *N*-terminal domain and an α-helix-rich *C*-terminal domain (Figure 2A). The *N*-terminal domain shows a high similarity to that of other RIPs. The *C*-terminal region includes a two stranded antiparallel β-sheet element connected by a short loop between the β7 and β8 strands; this structural motif is shorter than many other RIPs and may contribute to an increased accessibility to the substrate [17,18].

The active site is somewhat conserved among saporin isoforms. In saporin-S6, the residues proposed to be part of the active site cleft are Tyr^72^, Tyr^120^, Glu^176^, Arg^179^ and Trp^208^ (Figure 2B) [19]. The residues Glu^176^, Arg^179^ and Trp^208^ cover the same position of other RIP three-dimensional structures, and Tyr^72^ assumes a different side chain conformation. This conformation is very important for RIP activity because this residue has been shown to be responsible for the interaction with the target adenine [18]. Because the active site residues are somewhat conserved amongst saporin isoforms and other RIPs [20], differences in the activity could be attributed to amino acid differences lying outside the active cleft. The mutation of residue Asn^162^ to Asp^162^ (N162D) is sufficient to significantly reduce saporin-S6 *in vitro* activity and cytotoxicity. In fact, Asn^162^ lies in close proximity to three hydrophobic residues, Phe^149^, Ala^151^ and Val^153^. The negative charge of the N162D substitution may affect the stability of the active cleft by introducing a local structural change [21].

The analysis of the surface electrostatic potential (Figure 2C) indicates a negative potential (colored in red) in the active site region. Two glutamate residues (Glu^176^ and Glu^205^) are important for this negative charge. The small positive area (colored in blue) is due to the presence of only one arginine (Arg^209^) at the entrance and two (Arg^136^ and Arg^179^) inside the cavity of the active pocket [22]. 

Saporin-S6 is extremely resistant to high temperature, to denaturation by urea or guanidine and to attack by proteolytic enzymes [23]. Saporin-S6 is also very stable in response to chemical modifications such as those necessary for derivatization and conjugation procedures [24], and it is resistant to many freeze-thaw cycles (unpublished results from our laboratory). Altogether, these characteristics render saporin-S6 an interesting candidate for the construction of immunoconjugates.

### 2.2. Endocytosis and Intracellular Localization

Unlike type 2 RIPs, in which the presence of a lectin B-chain facilitates the endocytic mechanism, type 1 RIPs enter the cell with low efficiency. The cellular interaction of type 1 RIPs has been examined in many studies with inconclusive results. Particularly controversial is the debate regarding the mechanism of saporin-S6 endocytosis. It was initially suggested that saporin-S6, like all type 1 RIPs, enters cells through passive mechanisms such as fluid phase pinocytosis [25]. Saporin-S6 uptake by cells was described to occur by a mechanism that does not depend on specific binding sites [26]. However, the observations that some cell types show a moderate resistance to saporin-S6 cytotoxicity and that some organs are more sensitive to saporin-S6 intoxication led some researchers to search for a possible receptor. Receptor-mediated endocytosis through the α_2_-macroglobulin receptor, also called low-density lipoprotein receptor-related protein (LRP), was proposed as the binding mechanism for saporin-S6 [27]. A discrepancy was reported between the level of LRP and saporin-S6 cytotoxicity; *i.e.*, LRP-positive or -negative cell lines showed similar sensitivities towards saporin-S6, suggesting a receptor-independent endocytosis mechanism [28]. 

Recently, electron microscopy experiments were performed, indicating that saporin-S6 endocytosis by HeLa cells mainly occurs through non-coated vesicles [29]. After initial internalization, saporin-S6 should reach its cytosolic targets to exert its cytotoxic activity. Most information on the intracellular trafficking of RIP was obtained via studies of the endocytic pathway of ricin. Once it enters the cell, ricin travels backward from the Golgi complex to the endoplasmic reticulum (ER), where the separated A-chain exploits the ER-associated degradation pathway to enter the cytosol [30]. A comparison between the endocytosis of ricin and saporin-S6 by Vero or HeLa cells was performed using immunofluorescence and treatment with brefeldin A or chloroquine, indicating that the type 1 RIP follows a Golgi-independent pathway to the cytosol and does not require a low pH for membrane translocation [31]. Chloroquine and monensin have been used to raise the pH of the endosomal compartment to evaluate the intracellular routing of saporin-S6 in a CD30^+^ cell line [32] and in a prostatic cancer cell line [33]. Because these substances had no effect on the cytotoxicity induced by saporin-S6, unlike that induced by ricin, the intracellular transport of saporin-S6 to the cytosol should not involve lysosomes or the Golgi cisternae. In addition, the translocation mechanism should be low-pH independent [32,33]. There is evidence of saporin-S6 localization within the ER, Golgi apparatus and nucleus in HeLa cells, indicating that saporin-S6 can reach various intracellular compartments, possibly by more than one pathway. Saporin-S6 was found localized intracellularly within 20 minutes of exposure. Double immunofluorescence analysis performed by confocal microscopy showed that approximately 30% of saporin-S6 colocalized with the ER marker BiP, and approximately 7% co-localized with the Golgi apparatus, which was marked with GM130 [29]. Using transmission electron microscopy, after 20 min of incubation, gold-saporin-S6 molecules were seen mainly localized on the plasma membrane and in clear vesicles and vacuoles. Single gold particles were observed in sub-plasmalemmal clear vacuoles and free in the cytoplasm. After 40 min, saporin-S6 molecules were clustered in late endosomes and lysosomes in proximity to the nucleus and close to the rER cisternae. After 60 min of incubation, the nuclear localization of saporin-S6 was observed in approximately 10% of HeLa cells, with a gold labeling intensity ranging from moderate (8% of cells containing <10 gold particles/nucleus) to intense (2% of HeLa cells showing >10 gold particles/nucleus) [29]. This study was the first report of saporin-S6 detection in cell nuclei. It must be underlined that these results were obtained with HeLa cells and they cannot automatically be extended to any other cell type. The nuclear localization of saporin-S6 has been postulated by another recent paper in U266 and IM-9 cells [34]. However, in this paper, the RIP was biotinylated and delivered as a complex with an antibody-avidin fusion protein, which could have altered the intracellular trafficking of the toxin as compared to that of saporin-S6 alone.

### 2.3. Mechanisms of Intoxication and Cell Death Induced by Saporin-S6

Various biological assays have been used to assess the ability of saporin-S6, both free and conjugated, to cause necrosis and apoptosis in pre-clinical models of human cancers. In particular, the replicative capability of tumor cells can be detected by thymidine incorporation or clonogenic assays; the cell viability can be evaluated by many tests, such as protein synthesis inhibition, tetrazolium salts reduction, ATP measurement, LDH release and many others. Apoptosis *versus* necrosis can be detected by annexinV/propidium iodide staining. Activation of caspases, TUNEL, a variation of mitochondrial membrane potential and nuclear morphology, are the most common methods to detect apoptosis. The evaluation of these parameters in pre-clinical studies is essential in the design of ITs in order to increase apoptotic cell death, thus reducing the side effects triggered by the inflammatory response to necrosis. Moreover, these analyses may be useful in clinical trials to understand the efficacy and stability of the chimeric drugs. 

Experimental evidence has demonstrated the toxic effects of saporin-S6 both *in vivo* (in animal models) and *in vitro* (in many cell lines). In mice, an LD_50_ of 4.0 mg of RIP/Kg of body weight was calculated for saporin-S6. Intoxicated animals showed significant lesions in only three organs: the liver, spleen and kidney. After histopathological exam, the lesions revealed cell necrosis. Liver lesions were the most dramatic. Spleen lesions ranged from focal to more extensive necrosis of the red pulp. In kidney tissue, the proximal tubules were the most damaged [35]. No significant permanent lesions were observed after 14 days in mice intoxicated with saporin-S6 at non-lethal doses [36].

Most evidence about saporin-S6 cytotoxicity have been obtained by determining the inhibition of cellular protein synthesis or by determining the number of live (or dead) cells in comparison with untreated control cells by various viability/cytotoxicity tests. RIP activity is mostly expressed as the concentration that is able to inhibit 50% of protein synthesis (IC_50_). The IC_50 _ values for saporin-S6 reported in the literature can greatly vary (from 0.1 to 1000 nM), and the comparison between the numerous results available in literature is often difficult and arbitrary because of the heterogeneity of the experimental conditions, e.g., the type and/or number of cells used in the assays, the presence or absence of serum, the length of incubation with saporin-S6, the length of the experiment, *etc*. 

Initially, the toxic effects of saporin-S6 were attributed to the inhibition of protein synthesis, but the observation that RIPs also depurinate DNA and other nucleic acids opened new perspectives about the mechanism of cytotoxicity. The first descriptions that saporin-S6 is able to kill cells via apoptosis were reported in 1996 [37,38] (Figure 1). Some apoptotic features, such as chromatin fragmentation, apoptotic bodies and hypodiploid cells, were found in lymphocytes and in many hematological cancer cell lines [37,38]. Caspase-dependent apoptosis in U937 cells was reported to be induced by saporin-S6 through a mitochondrial cascade, independently of translation inhibition. In this study, saporin-S6 was found to induce apoptosis via the intrinsic pathway and the activation of caspase 9; the absence of the activation of caspase 8 and truncated Bid could indicate that in this model the extrinsic pathway is not involved in mediating saporin-S6-induced apoptosis [39]. Instead, in L540 lymphoma cells, both caspase 8 and 9 are activated by saporin-S6. Caspase inhibitors of extrinsic (Z-IETD) and intrinsic (Z-LEHD) pathways as well as the pan-caspase inhibitor Z-VAD and the necroptosis inhibitor necrostatin-1 caused the partial rescue of L540 cells from death by saporin-S6. These results indicate that apoptosis is the main death pathway in cells intoxicated with saporin-S6, but it is not the only pathway [40]. Indeed, the cell killing mechanisms of RIPs are in part independent of caspase activation at least at the IC_100_ (concentration inhibiting 100% of protein synthesis), which was the concentration used in those experiments [40]. In the U87 glioblastoma cell line, the activation of ERK1/2 has been observed to forego caspase 8 or 9 activation after saporin-S6 treatment. This ERK1/2 activation might induce a cell cycle arrest in G1 phase with a decrease in D1 cyclin levels. Furthermore, the inhibition of protein synthesis could activate p53 [41]. 

The capability of saporin-S6 to induce apoptosis results not just from its ability to block protein synthesis, as different mutants lacking RIP activity can induce DNA fragmentation and apoptosis [42]. In 2008, it was reported that the onset of apoptosis is independent of protein synthesis inhibition [39]. 

**Figure 3:**
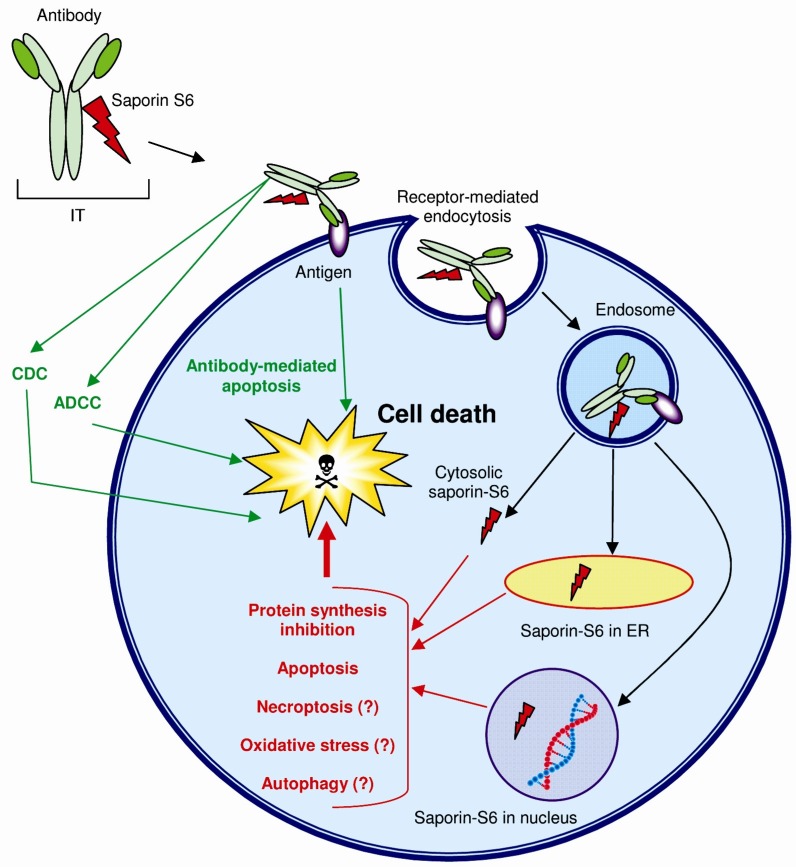
Multiple cell death pathways induced by saporin-S6 containing immunotoxins (ITs). The scheme shows the broad range of cell death mechanisms triggered by ITs. Once Saporin-S6 reaches the cytosol or ER or nucleus it can cause apoptosis activation (both caspase-dependent or -independent apoptosis), autophagy, necroptosis, oxidative stress and the inhibition of protein synthesis (in red). Moreover, cell death can also be activated by the antibody (in green) occurring through apoptosis or, when full-length antibodies are used through complement-dependent cytotoxicity (CDC) and antibody-dependent cellular cytotoxicity (ADCC).

To unify the intriguing results printed above, the following hypothesis may be put forward. Cell death by saporin-S6 may be induced by various cell injuries, including the inhibition of protein synthesis through RIP activity and DNA damage, either via *N*-glycosylase activity or as a result of oxidative stress-induction [34], or by other types of damage to the cell machinery that leads to apoptosis or different types of cell death (*i.e.*, autophagy or necroptosis) (see Figure 3). In particular, the activation of the autophagic pathway may promote cell death as a result of cellular atrophy or leading to the execution of apoptotic or necrotic cell death programs [43].

The presence of saporin-S6 in the nucleus suggests that DNA damage could be one of the mechanisms used by this protein, and possibly other RIPs, to kill the cell, specifically by inducing DNA-dependent apoptotic death. In HeLa cells, a specific comet assay that detects DNA gaps resulting from the detachment of purinic/pyrimidinic bases was developed to investigate the possibility that saporin-S6 can reach the nucleus and exert its activity on DNA. The addition of endonuclease IV enabled the repair of nuclear damage in most cells, highlighting that the repair of DNA damage caused by base cleavage could be induced by the deadenylation activity of saporin-S6 [29].

A global gene expression analysis of two human malignant B-cell lines revealed that an IT containing saporin-S6 could upregulate eleven genes involved in the cellular response to oxidative stress/DNA damage (*KLF6*, *TXNIP*, *NFKBIE*, *CDC14B*, *BHLHB2*, *GADD45B*, *HIST2H4*, *TSC22D3*, *RGS1*) or involved in mRNA processing (*THUMPD2*, *FYTTD1*) [34]. The authors suggested that saporin-S6 induces in cells a transcriptional response dependent on oxidative stress/DNA damage, resulting in signal transduction blockage, cell cycle arrest and, consequently, apoptosis. 

In conclusion, it is likely that the cell damage induced by saporin-S6 represents a cell type-dependent multi-direction pathway in which apoptosis seems to be the main detectable effect in several different cell lines. For this reason, a better understanding of the molecular mechanism of saporin-S6 intoxication could be helpful to designing an optimal therapeutic application. In fact, the possibility of modulating neoplastic cell killing through different ways adds new opportunities to develop combined synergistic immuno- and chemo-therapy, overcoming the problem of drug resistance.

## 3. Immunotoxins

When toxins are conjugated to targeting proteins, they can be specifically delivered toward unwanted cells responsible for diseases. Antibodies and their fragments have been the most utilized carriers, but other molecules have also been employed, such as hormones, growth factors, antigens, cytokines and others [44]. The term “immunotoxin” generally refers to a toxin targeted by an antibody, either intact or a fragment, whereas toxins linked to other carriers are more commonly referred to as “chimeric toxins” or simply “conjugates”.

To date, ITs have provided excellent results in hundreds of different models in pre-clinical studies [6,11,45] and in clinical trials [12,46]. The best results have been obtained in cancer therapy, especially in hematological malignancies. The efficiency of an IT in killing the target cells mainly depends on the cell type, antigen density, binding affinity and intracellular routing.

The main reported undesirable consequences of ITs in clinical therapy are immunogenicity, and vascular leak syndrome (VLS). Anti-toxin antibodies are produced by approximately 90% of epithelial cell cancer patients after 1 or 2 cycles of treatment with ITs [9]. However, patients with hematologic cancers show a lower incidence of anti-toxin antibodies, which is detectable only after several cycles of therapy [9]. This occurs because end-stage onco-hematological patients are often heavily immunosuppressed. VLS is caused by an increase in vascular permeability due to endothelial cell damage. VLS is associated with an extravasation of fluids into tissues with consequent edema, hypotension and, in severe form, signs of pulmonary and cardiovascular failure [47].

Despite the presence in literature of a large number of studies with ITs containing different toxic moieties (plant and bacterial toxins, enzymes, drugs, *etc*.), the published results are often not comparable each other because of the difference in the carrier moiety, the target cells and the experimental conditions, also when ITs are directed to the same target molecule. When ITs containing different type 1 RIPs or the ricin A chain linked to the same antibody were compared in the same *in vitro* experiment, those containing saporin-S6 always resulted the most, or one of the most effective [11].

The use of saporin-S6-containing ITs to treat hematological tumors often revealed a certain grade of toxicity for bone marrow, due to the expression of the targeted antigen on various normal, hematopoietic progenitor cells. However, in different studies it has been demonstrated that although the ITs may be cytotoxic against committed progenitor cells, these cells can be repopulated by the pluripotent progenitors that are not affected by treatment with the conjugates [34,48,49].

Additionally, saporin-S6 has been utilized in neuroscience studies in which it has been conjugated to specific molecules (e.g., substance P, anti-NGFR antibodies) for the purpose of selectively destroying neurons. The permanent and selective killing of neurons is an important model for the study of behavior, neuronal loss (e.g., Alzheimer’s disease), *etc*. Several studies were performed in animals with saporin-S6-containing ITs that aimed to suppress some forms of strong chronic pain through the permanent removal of a small number of spinal neurons, which transmit chronic pain signals, without affecting the sensitivity to acute pain. Despite the importance of these studies, they are not a topic of the present review, and thus we refer the reader to specific reviews on the subject (e.g., [50,51]).

### 3.1. Chemical and Recombinant Immunotoxins

First-generation RIP-based ITs were produced by chemically coupling native toxins to antibodies by the formation of disulfide bonds that connect the toxic moiety to the carrier. Chemically coupled ITs have several advantages: they are relatively simple to produce at high yields, and they have good stability. The main disadvantage is represented by their heterogeneous composition. 

A new generation of ITs was produced using recombinant DNA techniques, and they are called recombinant ITs. They are obtained by linking the gene portion encoding the antigen-binding fragments of an antibody (Fab or Fv) to the gene encoding the native catalytic domain or a mutated version of the toxin. The resulting construct can be expressed in various hosts, such as bacteria [52], yeast [53] or algae [54]. The first generated recombinant ITs utilized single-chain variable fragments (scFvs) as carrier moiety, but they were then substituted by disulfide-stabilized Fvs (dsFvs). In the scFv fragment, the antigen-binding portion of the heavy-chain variable region is connected by a short linker to the variable region of the light chain [55], but in dsFvs the peptide linker was replaced by a disulfide bond, which confers them enhanced stability [44]. Moreover, a panel of new partially or fully humanized antibodies was employed as a carrier moiety to minimize the risk of immunogenicity and toxicity in patients.

### 3.2. Immunotoxins Containing Saporin-S6

The remarkable resistance to denaturation, proteolysis and chemical modifications as well as its high enzymatic activity and low cytotoxicity as an unconjugated molecule (as detailed in Section 2) suggests that saporin-S6 represents an ideal candidate for the construction of conjugates for therapeutic use. In 1985, saporin-S6 was conjugated for the first time to the murine anti-Thy 1.1 mAb (OX7) and to its F(ab’)_2_ fragment [56]. In 1989, the cytotoxicity of the OKT1(murine)/saporin-S6, an anti-CD5 IT, was evaluated on fresh chronic B lymphocytic leukemia cells from 31 patients [57]. Since then, saporin-S6 has been largely used as a toxic moiety in a variety of immunoconjugates targeting different malignant hematological cells and solid tumors, with promising results in several pre-clinical studies. Hematological cells represent the best candidate for targeted therapy because they offer surface target antigens that are easier to access *in vivo* than do solid tumor cells. 

In this section, we describe the experiments that have been carried out with saporin-S6-containing ITs. The paragraphs are organized based on the origin of the utilized antibody, that is, murine, chimeric, humanized or scFv. For each category, the results are grouped according to the recognized molecular target, treating both hematological and solid tumors. For a more complete review of saporin-S6-containing ITs, we direct the reader to the review by Polito *et al*., 2011 [12]. 

#### 3.2.1. ITs Containing Saporin-S6 and Murine mAbs (and Other First Generation Abs)

Saporin-S6 was first conjugated to murine anti-CD2 mAbs (GT2, OKT11, 8E5B3, 8G5B12 and 7A10C9) to produce several ITs that showed an increased cytotoxicity compared to native saporin-S6. Anti-CD2 conjugates evaluated *in vitro* were found to inhibit protein synthesis with IC_50_s ranging from 0.1 to 100 pM [58]. Similar *in vitro* efficacy was observed for an anti-CD7 HB2/saporin-S6 IT, which had an IC_50_ in the pM range. When tested in a mouse model of human T-cell leukemia, this IT showed a therapeutic effect with a single intravenous (i.v.) dose of 10 µg of IT (0.5 mg/kg), achieving 50% survival at day +150 [59]. Further studies on this IT provided additional interesting information about the *in vivo* therapeutic efficacy of HB2/saporin-S6. In fact, the conjugation of saporin-S6 to an HB2 F(ab’)_2_ fragment, which is incapable of recruiting NK cells, led to the lowered *in vivo* efficacy of the IT [60]. A decreased activity of HB2/saporin-S6 was also shown in the treatment of a NOD/SCID mouse leukemia model, which has reduced cytolytic NK activity [61]. Taken together, these findings suggest that host-mediated antibody-dependent cell cytotoxicity (ADCC) positively contributes to HB2/saporin-S6 efficacy. Further attempts to improve the *in vivo* efficacy of this IT, both with the insertion of hindered or non-hindered disulfide cross-linkers or the coupling of one or two saporin-S6 moieties per IT molecule, failed to achieve significant differences in terms of pharmacokinetic and therapeutic effects [62,63]. The BU12 anti-CD19 mAb covalently coupled to saporin-S6 showed an IC_50_ in the nM range for the CD19^+^ B-cell acute lymphoblastic leukemia cell line NALM-6. In SCID mice injected with NALM-6, BU12 IT administration (3 × 10 µg total, each injection being given i.v. on alternate days, *i.e.*, 7, 9 and 11 days after tumor cell injection) led to a significantly prolonged survival *versus* sham-treated controls, with 40% of animals alive and disease free at +110 days [64]. A very strong *in vitro* efficacy was observed with saporin-S6 conjugated to the anti-CD22 mAb OM124, with IC_50_s ranging from 0.001 to 10 pM. The *in vivo* treatment of SCID mice bearing transplanted Daudi cells with 0.5 mg/kg of OM124/saporin-S6 given at days +1, +4, +7 after tumor injection resulted in 33% of the animals being tumor-free at day +220. The combination therapy with cyclophosphamide (60 mg/kg) given on days +1 and +2, and the administration of this IT on days +1, +4, +7 showed a better efficacy with 66% of the animals being tumor-free at day +220 [65]. 

The efficacy of an anti-CD38 IB4/saporin-S6 was evaluated in different CD38^+^ human cell lines and in CD38^+^ malignant cells from a non-Hodgkin lymphoma (NHL) patient. All of the cell lines tested were found to be very sensitive to this IT, with IC_50_s in the pM range. The neoplastic CD38^+^ cells obtained from the NHL patient were completely eliminated with IB4/saporin-S6 at 10 nM concentration [66]. IC_50_s lower than 10 nM were observed *in vitro* for the anti-CD80 B7-24/saporin-S6 in CD80^+^ B-cell line, Raji, and in Reed-Sternberg cell lines HDLM2 and KM/H2 [67]. The anti-CD80 mAb M24 conjugated to saporin-S6 exhibited strong cytotoxicity against Raji and L428 cell lines, with IC_50_s in the pM range. In the same experiments, an anti-CD86 1G10/saporin-S6 IT was almost 1 log less toxic than M24/saporin-S6 [49]. Two plasma cell-reactive mAbs, B-B2 and B-B4, were conjugated to saporin-S6 for the immunotherapy of multiple myeloma. Both of these ITs were found to be suitable for *ex vivo* bone marrow purging and showed no reactivity with hemopoietic precursor cells. B-B4 was the more effective of the two conjugates [68]. 

The mAb Ber-H2 was conjugated with saporin-S6 giving an IT that specifically inhibits protein synthesis in Hodgkin-derived cell lines with IC_50_s ranging from 0.01 to 1 pM [69]. In a SCID mouse model of human xenografted CD30^+^ anaplastic large-cell lymphoma (ALCL) (JB6 cell line), a 3-day treatment with non-toxic doses of Ber-H2/saporin-S6 (50% of LD_50_) induced complete remission in 80% of mice when treatment started 24 h after tumor transplantation, and 30% of complete remission when started at a later stage (40–60-mm^3^ tumor volume) [70]. Strong anti-tumor activity of the same IT was also observed in another SCID mouse model of ALCL (D430B cell line). A dose of 0.1 mg/kg IT given 48 h after tumor transplantation caused complete remission in 4/6 mice and partial remission in 2/6 mice [71].

The polyclonal antibody ATG Fresenius-S was prepared by immunizing rabbits with non-fractionated human thymocytes isolated by Ficoll density gradient centrifugation. ATG was conjugated to saporin-S6, and the resulting IT showed a strong cytotoxic effect on lymphoma- and leukemia-derived cell lines. This IT enhanced saporin-S6 toxicity by approximately 2–3 logs and induced a time-dependent activation of caspase-3/7 in Raji cells [72].

Unlike malignant hematological cells, ITs reach solid tumor cells poorly, mainly due to non homogeneous cancer vasculature and high interstitial pressure that obstacle IT diffusion into the cancer mass. Moreover, other obstacles are represented by the less availability of cancer specific antigens and often the worse cellular internalization of the immunocomplex in solid tumors respect than hematological ones. For these reasons, ITs often showed good *in vitro* anti-tumor efficacy on solid tumors, but in animal models they gave less resounding results than those described on hematological tumors [11,12]. Several ITs have been obtained by conjugating saporin-S6 to murine mAbs that recognize antigens on solid tumor cells. Ep2/saporin-S6 IT, which recognizes the high molecular weight melanoma-associated antigen showed an *in vitro* IC_50_ of approximately 0.1 nM, while it did not affect antigen-negative melanoma cells at 100 nM dose [73]. Loco-regional administrations (e.g. intravesical, intrathecal, *etc*. applications) could be useful to by-pass most IT disadvantages. The 48-127/saporin-S6 IT recognizing gp54, a glycoprotein that is expressed on all human bladder tumors, showed an *in vitro* IC_50_ in the nM range on the target T24 cell line. The authors demonstrated that a 2 h incubation of the cells with the IT was sufficient to ensure optimal binding and endocytosis, thus the IT maintained the complete anti-tumor efficacy in a time compatible with bladder irrigation [74]. Tumor cells generally express higher level of transferrin receptor (TfR) than normal tissues, this led many researchers to utilize TfR as target for immunotherapy. The efficacy of an anti-TfR murine mAb conjugated to saporin-S6 was compared to transferrin/saporin-S6 conjugates. Both conjugates were found to be equally effective with IC_50_ in 0.1 nM range, but the transferrin/saporin-S6 conjugate, although presenting a minor risk with regard to immunogenicity, was influenced *in vivo* in its efficacy by free transferrin and iron saturation, whereas the anti-TfR/saporin-S6 IT was not as subdued by these influences [75]. The *in vivo* efficacy of an anti-receptor protein tyrosine phosphatase β mAb conjugated to saporin-S6 was evaluated in athymic nude mice bearing the U87 glioblastoma cells. The IT was given intrathecally once tumors had reached a mean tumor volume of 130 mm^3^ at doses of 15 and 30 µg twice a week for two weeks, the IT was able to slow down tumor growth by 25% and 73%, respectively. The medium survival time of mice was prolonged from 18.6 days (PBS) to 32.1 (IT 30 µg). The IT did not cross-react with the investigated tissues (colon, kidney, liver, small intestine, and stomach) [76].

#### 3.2.2. Immunotoxins Containing Saporin-S6 and Chimeric mAbs

Chimeric antibodies have been developed in order to eliminate the human anti-mouse antibody response seen in patients treated with murine antibodies. The chimerization process involves the replacement of mouse constant domains with human constant domains, thereby eliminating the immunogenic portions without altering the specificity for the target.

Rituximab is an anti-CD20 chimeric mAb that is widely used as single agent for the treatment of patients with CD20^+^ NHL or chronic lymphocytic leukemia. In 1997, Rituximab was approved by the US FDA for the treatment of recurrent/refractory follicular NHL and of untreated aggressive NHL in combination with the cyclophosphamide-hydroxydaunorubicin-oncovin-prednisone (CHOP) regimen [77]. Rituximab/saporin-S6 was the first IT in which saporin-S6 was conjugated to a chimeric mAb. This IT exhibited strong cytotoxicity in target cells and inhibited 50% of protein synthesis at a concentration of approximately 0.2 nM. The conjugate also induced apoptosis in 95% of treated cells at a concentration of 10 nM. The cytotoxicity of Rituximab/saporin-S6 was augmented by the co-administration with the chemotherapy drug fludarabine, leading to the complete elimination of the malignant population [78].

The anti-hTfR IgG3-Av, consisting of a mouse/human chimeric IgG3 genetically fused to chicken avidin, was conjugated to biotinylated saporin-S6. This IT resulted strongly cytotoxic in myeloma cells expressing different levels of TfR, activating multiple cell death pathways simultaneously [79].

The ch25A11/saporin-S6 IT specifically recognizes the CUB domain-containing protein 1 that is expressed by prostate cancer cells. This IT was evaluated *in vivo* in SCID mice bearing prostate carcinoma cells and demonstrated a fairly good anti-tumor activity. The dose-finding study showed that the optimal regimen for mice bearing PC-3 cells (3 × 10^6^) injected with ch25A11/saporin-S6 at 0.4 mg/kg was a three-dose treatment on days +7, +10, and +17. The IT caused acute toxicity, shown by the acute, but reversible body weight loss 1 day after injection, but all the mice recovered their weight after the treatment. Tumor growth was inhibited by approximately 65% by IT only when administered i.v., whereas the IT inhibited tumor metastasis when it was administered both i.v. and subcutaneously [80].

#### 3.2.3. Immunotoxins Containing Saporin-S6 and Humanized mAbs

Further improvements of chimeric mAb immunogenicity and efficacy have led to the construction of humanized mAbs in which only the hypervariable regions are of murine origin. 

Epratuzumab is a humanized mAb that selectively binds the CD22 antigen. The Epratuzumab/saporin-S6 IT showed strong *in vitro* cytotoxic effects demonstrated by the complete loss of viability, the total inhibition of protein synthesis and the induction of apoptosis in CD22^+^ target cell lines at concentrations of approximately 0.1–1 nM. This IT showed potent anti tumor activity in a SCID mouse Raji xenograft model of aggressive human lymphoma [81,82]. The anti-CD22 IT HB22.7/saporin-S6 exhibited strong cytotoxicity in NHL cell lines showing IC_50_ values in 10 pM range and completely prevented tumor development in athymic nude mice when the treatment was started within 24 hours from tumor inoculation, whereas the mixture of free HB22.7 and saporin-S6 did not exert cytotoxicity [83]. Moreover, in NOD/SCID xenograft mice, the IT increased the median survival time from 20 (control mice) to over 50 days (treated mice) [84].

With regard to solid tumors, the humanized biotinylated mAb hJ591 (anti-PMSA) was conjugated to streptavidin-saporin-S6, and its cytotoxic effects were evaluated in prostate cancer cells and in an LNCaP xenograft model in athymic nude mice. In cell lines, the IT showed IC_50_ values in the nM range and induced apoptosis in 40%–60% of target cells. The IT caused a reduction in tumor growth in treated mice by approximately 5- to 6-fold with respect to the control mice [85].

#### 3.2.4. Immunotoxins Containing Saporin-S6 and Single-Chain Variable Fragments

Variable fragments are the smallest functional units of antibody molecules required to maintain the structure and specificity of the whole antibody. Because of their small sizes, scFv containing ITs can access solid tumors more easily than ITs containing whole mAbs. However, the scFvs have also some disadvantages as the loss of CDC and ADCC activities, due to the lack of the Fc portions. Moreover they have shorter half lives in blood than the whole antibodies.

Treatment with the anti-CTLA-4 scFv-83/saporin-S6, scFv-67/saporin-S6 and scFv-40/saporin-S6 ITs resulted in specific cytotoxicity against activated T lymphocytes and for several target cell lines [86]. The scFv-83/saporin-S6 IT was able to induce apoptosis at a concentration of 10 nM in more than 90% of treated acute myeloid leukemia patient cells [87]. This IT was also tested in a model of tumor rejection consisting of C57BL/6 mice bearing a murine H.end endothelioma cell line derived from DBA/2 mice. Lymphoid infiltration was notably reduced, demonstrating that this IT was active *in vivo*. The toxicity of the IT I/F8 scFv/saporin-S6 was assayed on both human and murine ALCAM/CD166^+^ cell lines, and toxicity was at least 100-fold higher than that of free saporin-S6 with IC_50_s in the nM range [88]. 

#### 3.2.5. Saporin-S6-Containing Immunotoxins in Clinical Trials

The few saporin-S6-containing ITs that have entered clinical trials so far have generated very interesting results. Below, we summarize the main results obtained in the treatment of hematological malignancies. 

The first phase I/II clinical trial with a saporin-S6-containing IT was carried out in 1992 using the anti-CD30 Ber-H2/saporin-S6 IT (0.2 mg/kg, as RIP, in one or two weekly doses i.v.) in four patients with advanced refractory Hodgkin’s disease (HD), achieving promising results with only mild and transient side effects. A reduction in tumor mass was observed in all patients, with 75% of them achieving partial remission and 50% displaying a complete relief of systemic symptoms with responses lasting 6–10 weeks [89]. The enrollment of twelve patients with advanced HD extended the same trial. The final results of this study included a tumor mass reduction in 60% of cases, with partial remission in approximately 42% of patients and a minor response in 17%. Responses lasted between 2 and 4 months [90].

Side effects observed in about 70% of patients included fever, myalgias, grade I VLS, thrombocytopenia and a transient increase in transaminases. The maximum tolerated dose, as defined by a grade III reversible VLS, was reached in one patient at dosage of 0.2 mg/kg as saporin-S6 (0.8 mg/kg as IT), given as single administration. Some of the IT related symptoms, such as fever and myalgias, were abolished by concomitant steroid therapy. In all patients, Abs against both the murine mAb and the toxin were detected 2–4 weeks after the treatment [89,90].

Bispecific F(ab’)_2_ antibodies (4KB128 and HD6) were employed to target saporin-S6 to the CD22 surface antigen in the treatment of lymphoma in two Phase I/II clinical trials. One patient with end-stage NHL received a total of 5 mg of saporin-S6 complexed with a pair (50 mg) of anti-CD22 bispecific Abs over 15 days (0, +7, +15), achieving a complete clearance of the tumor from the blood, clearance of the ascites and shrinkage of the tumor masses. The patient showed no sign of an anti-saporin-S6 response, but developed a strong anti-mouse Fab response 28 days after the treatment began. In the same trial another patient with end-stage chronic lymphocytic leukemia, treated with the bispecific F(ab’)_2_ antibody RFB-9 targeting saporin-S6 to the CD19 antigen (total of 10 mg of saporin-S6 complexed to 100 mg of anti-CD19 bispecific Ab, at days 0, +7, +28, +42), showed no therapeutic effect over 45 days. Neither patient experienced any toxic side-effects [91]. Five low-grade, end-stage, B-cell lymphoma patients received between 3 and 6 doses of the above described anti-CD22 ITs with weekly infusions containing escalating doses of the complexes (ranging from 1 to 4 mg/dose, for a total of 5–20 mg, as RIP).

Only grade I toxic effects were seen, with mild fever, weakness and myalgia for 1–2 days after the treatment. All weekly treatments were well tolerated; the only significant toxicity was slight inflammation of the vein used for infusion (2/5), some myalgias and general weakness for 24 to 48 h after the treatment. There was no evidence of weight gain or other signs of VLS. Only one patient developed an antibody response to the infused complexes, both against antibody and saporin-S6. All patients showed rapid and significant responses to the treatment, and all showed at least a 50% reduction in measurable disease even if no response persisted for the 28 days necessary for the definition of a partial response [92,93]. 

#### 3.2.6. New Strategies to Improve Saporin-S6 Immunotoxins in Cancer Therapy

Some efforts have been made to enhance the toxicity of saporin-S6 alone or as ITs in order to develop a new therapeutic approach for the treatment of neoplasms, especially solid tumors. A further improvement of saporin-S6-based therapy could lead to the better design of ITs and related delivery strategies to improve saporin-S6-based anti-tumor therapy. 

One of the strategies employed to enhance IT cytotoxicity is the photochemical internalization (PCI) of therapeutic molecules. PCI is a drug delivery method for the cytosolic release of drugs from the endocytic compartment. The method takes advantage of the use of photosensitizers that localize to the membrane of the endocytic vesicles and their ability to cause membrane breakage and the subsequent release of entrapped drug into the cytosol after controlled light exposure. As ITs are taken up by receptor-mediated endocytosis [1], the combination of targeted therapy with the PCI technology, during which light-activation of the drug is constrained to the tumor, should be particularly beneficial. In fact, this strategy provides a wider therapeutic window for the targeting drug and should improve efficacy, especially in the treatment of solid tumors. It has been shown that PCI synergistically enhances the cytotoxicity of cetuximab/saporin-S6, an IT targeting EGFR, which consists of the chimeric mAb cetuximab bound to saporin-S6 by a biotin-streptavidin linkage. The PCI treatment enhanced the cytotoxicity of the IT in a synergistic manner in EGFR-expressing carcinoma cell lines derived from different tumor tissues [94]. PCI was also found to enhance the toxicity of anti-HER2 trastuzumab/saporin-S6 IT on trastuzumab-resistant HER2+ Zr-75-1 cells, even though a higher concentration of this IT was required to achieve a similar cytotoxic effect with respect to cetuximab/saporin IT (100 pM *versus* 3 pM, respectively) [95]. In addition, PCI was found to be an efficient method for the selective killing of CD133^+^ cancer cells that have cancer stem cell properties and are resistant to photodynamic therapy. PCI of the anti-CD133 saporin-S6-containing ITs efficiently depleted sarcoma cells at fM concentrations, leading to a reduced sarcoma tumor-initiating capacity. Sarcoma cells with stem cell properties were also subjected to sub-toxic treatments to allow the transplantation of viable cells in NOD/SCID IL2Rγ mice, confirming the reduced tumor-initiating capacity after IT treatment [96,97].

Another improvement of saporin-S6-based ITs results from their combination with a mixture of saponins, which are in general tenside-like compounds able to interact with cholesterol within membranes. This combination was found to enhance saporin-S6 toxicity [98], so the employment of saponins has been used to ameliorate the intracellular uptake of saporin-S6-based ITs in solid tumors. The combined application of saponins with the hEGF/saporin-S6 IT showed a 6900-fold enhancement in the *in vitro* cytotoxic efficacy against target cells. *In vivo* studies in over-expressing, EGFR-tumor bearing mice showed a reduction in the average tumor size of more than 90% as a result of this combined therapy [99,100]. 

Another approach to selectively target saporin-S6 to malignant cells is to encapsulate the toxin into targeted nanoparticles, such as liposomes, nanoemulsions, solid lipid nanoparticles, polymeric nanoparticles, dendrimers, carbon nanotubes and magnetic nanoparticles. The size of particles used for medical purposes is preferably in the 5–200 nm range. Their conjugation to mAbs allows the site-directed delivery of drugs toward specific targets. Saporin-S6 was loaded into targeted cationic liposomes and PEG-liposomes, and their cytotoxicity was evaluated *in vitro*. These conjugates showed enhanced toxicity with respect to saporin-S6, especially in combination with the PCI technique [101].

## 4. Conclusions

The chemico-physical and structural properties of the type 1 RIP saporin-S6 allow for understanding some reasons for its success as a toxic moiety of ITs. First, the accessibility of the active site to the substrate could justify the high enzymatic activity; second, the high resistance to proteolysis surely contributes to the stability of the enzyme. Indeed, saporin-S6 shows good resistance to blood and endosomal proteases. Moreover, it well maintains its properties after chemical modifications and conjugation processes. In addition, studies on cell interaction and intracellular routing have shown the low level of unspecific toxicity, due to the scarce ability of the saporin-S6 to enter the cell in absence of a specific carrier. On the other hand, once the endocytosis is facilitated, this RIP may reach many cellular compartments, where it appears to have a wide range of possible intracellular substrates, thus triggering multiple cell death pathways, rendering impossible the selection of RIP-resistant mutants (Figure 3).

Because of all the characteristics reported above, saporin-S6 could represent the ideal toxic moiety to obtain conjugates, as also demonstrated by the numerous publications reporting interesting results (Table 1). In fact, saporin-S6 has been utilized to construct conjugates and ITs against several targets in many pre-clinical studies, leading to promising outcomes in most cases. The great efficacy has been reported in different models of hematological tumors. In the experiments conducted on mice, treatment with saporin-S6-containing ITs was able to strongly reduce the size of transplanted tumors in all cases, and in several models, completely eliminate tumor masses. Moreover, saporin-S6 ITs have a multifaceted cytotoxicity due to the combined death mechanisms of both the antibody and RIP.

**Table 1:** Summary of the main saporin-S6 containing ITs.

**Hematological Tumors**				
**Antibody**	**Origin**	**Antigen Target **	**Tumor**	**Ref** **erence**
GT2, OKT11, 8E5B3, 8G5B12, 7A10C9	Murine	CD2	T-cell chronic lymphocytic lymphoma	[58]
OKT1		CD5	B lymphocytic leukemia	[57]
HB2		CD7	T-cell acute lymphoblastic leukemia	[59]
BU12		CD19	B-cell lymphoblastic leukemia	[64]
OM124		CD22	B lymphoblastoid, Burkitt’s lymphoma	[65]
F(ab’)_2_ BsAb			Non-Hodgkin’s lymphoma	[91,93]
4KB128+ HD6			B-cell lymphoma	[92]
BerH2		CD30	Hodgkin’s disease, Anaplastic large-cell lymphoma	[69,70,71,89,90]
IB4		CD38	Non-Hodgkin’s lymphoma	[66]
B7-24		CD80	Burkitt’s lymphoma, Hodgkin’s disease	[67]
M24 + IG10		CD80/CD86	Burkitt’s lymphoma, Hodgkin’s disease	[49]
B-B2, B-B4		CD138	Multiple myeloma	[68]
ATG	Rabbit	Various	Lymphoma and leukemia cells	[72]
Rituximab	Chimeric	CD20	Non-Hodgkin’s lymphoma	[78]
Anti-hTfR IgG3-Av		TfR	Myeloma	[34,79]
Epratuzumab	Humanized	CD22	Non-Hodgkin’s lymphoma	[81,82]
HB22.7			Non-Hodgkin’s lymphoma, Acute lymphoblastic leukemia	[83,84]
scFv-83, scFv-67, scFv-40	scFv	CTLA4	Activated T lymphocytes, Myeloid leukemia	[86,87]
**Solid Tumors**				
**Antibody**	**Origin**	**Antigen Target **	**Tumor**	**Ref** **erence**
Ep2	Murine	HMW-MAA	Melanoma	[73]
48–127		gp54	Bladder tumor	[74]
42·6.3		TfR	Glioblastoma	[75]
7E4B11		RPTPbeta	Glioblastoma	[76]
ch25A11	Chimeric	CDCP1	Prostate carcinoma	[80]
Cetuximab		EGFR	Various	[94]
hj591	Humanized	PSMA	Prostate carcinoma	[85]
Trastuzumab		HER2	Breast carcinoma	[95]
I/F8	scFv	CD166	Various	[88]

The studies, summarized in this review, indicate that saporin-S6-based ITs are extremely powerful *in vitro* and maintain good anti-tumor effectiveness *in vivo*. Clinical results have demonstrated the efficacy of ITs in cancer patients who are refractory to traditional modalities of treatment, including surgery, radiation therapy and chemotherapy. 

The IT specificity is based upon characteristics (surface antigens) that are completely independent from the parameters determining the toxicity of chemo- and radiotherapy. This difference allows for a non-superimposition of side effects and for unimpaired cytotoxicity toward cell clones that are resistant to chemo- and radiotherapy. 

The large number of patents protecting immunoconjugates or their components indicates the fervid interest in the field by institutional researchers and pharmaceutical companies. We and many other investigators are confident that in the near future, immunoconjugates could have an important role in cancer treatment [9]. The greater than twenty antibody-drug conjugates and the eight ITs in clinical trials demonstrate the maturity of this approach [102]. A good example is represented by the anti-HER2 conjugate T-DM1 (trastuzumab emtansine), recently (February 2013) approved by the US FDA for patients with HER2-positive, late-stage breast cancer [102]. 

In the last years many clinical trials have been conducted with ITs, prevalently containing deglycosylated A chain of ricin, truncated diphtheria toxin or *Pseudomonas* exotoxin A [102]. RIP containing ITs present some advantages over those containing bacterial toxins, they lack the natural immunization often present against bacterial proteins and are numerous and often not immunologically related. The availability of different toxic proteins, together with the recent progresses in the production of non-immunogenic Abs (humanized mAbs or scFvs) could be useful to bypass the immunogenicity problem by sequential administration of ITs containing different non-cross reacting RIPs.
